# Home Care Resiliency during the COVID-19 Pandemic: Older Adult-Home Care Aide Dyads’ Perspectives

**DOI:** 10.1093/geroni/igab046.2674

**Published:** 2021-12-17

**Authors:** Naoko Muramatsu, Lijuan Yin, Maria Caceres, Jordan Skowronski

**Affiliations:** 1 University of Illinois Chicago, Chicago, Illinois, United States; 2 University of Illinois Chicago, University of Illinois Chicago, Illinois, United States

## Abstract

Homecare has increased its value as an alternative to nursing homes and adapted to evolving COVID-19 challenges. However, little is known about how COVID-19 has impacted community-dwelling older adults who need assistance with daily activities, including dressing, cooking, and shopping. Guided by the stress process framework, this mixed-method study examined how older homecare recipients experienced the acute and chronic stress during the first eight months of the pandemic, focusing on the role of home care aides (HCAs) in the context of Medicaid-funded in-home services. Thirty-five dyads of care recipients and HCAs participated in a COVID telephone survey as part of a larger study. Care recipients were typically older minority (40% African American, 31% Latinx) women (77%). Their COVID-related anxiety level, assessed by a 6-item Spielberger State Anxiety Inventory (1 “not at all” to 4 “very much”), was 2.2 (SD=0.9). While COVID-19 drastically reduced contacts with family members and healthcare providers, HCAs continued to provide care in person. One care recipient said, “Fortunately, I still have my HCA come and that keeps me sane.” HCAs showed resilience while facing their own family- and work-related stress: “I have followed the rules and just adapted. (COVID) did not affect the activities for my client.” Some dyads, however, experienced care disruptions because of COVID infection or fear in one or both parties. COVID-19 has demonstrated homecare resilience at the person-, dyad-, and organization-levels, calling for equitable, sustainable home-based care for a growing number of older adults who desire to stay in the home.

